# Diabetic Retinopathy Grading by Digital Curvelet Transform

**DOI:** 10.1155/2012/761901

**Published:** 2012-09-12

**Authors:** Shirin Hajeb Mohammad Alipour, Hossein Rabbani, Mohammad Reza Akhlaghi

**Affiliations:** ^1^Biomedical Engineering Department, Medical Image & Signal Processing Research Center, Isfahan University of Medical Sciences, Isfahan 81745319, Iran; ^2^Ophthalmology Department, School of Medicine, Isfahan University of Medical Sciences, Isfahan, Iran

## Abstract

One of the major complications of diabetes is diabetic retinopathy. As manual analysis and diagnosis of large amount of images are time consuming, automatic detection and grading of diabetic retinopathy are desired. In this paper, we use fundus fluorescein angiography and color fundus images simultaneously, extract 6 features employing curvelet transform, and feed them to support vector machine in order to determine diabetic retinopathy severity stages. These features are area of blood vessels, area, regularity of foveal avascular zone, and the number of micro-aneurisms therein, total number of micro-aneurisms, and area of exudates. In order to extract exudates and vessels, we respectively modify curvelet coefficients of color fundus images and angiograms. The end points of extracted vessels in predefined region of interest based on optic disk are connected together to segment foveal avascular zone region. To extract micro-aneurisms from angiogram, first extracted vessels are subtracted from original image, and after removing detected background by morphological operators and enhancing bright small pixels, micro-aneurisms are detected. 70 patients were involved in this study to classify diabetic retinopathy into 3 groups, that is, (1) no diabetic retinopathy, (2) mild/moderate nonproliferative diabetic retinopathy, (3) severe nonproliferative/proliferative diabetic retinopathy, and our simulations show that the proposed system has sensitivity and specificity of 100% for grading.

## 1. Introduction 

Diabetic Retinopathy (DR) is a leading cause of vision loss in the working class in the world [1, 2]. If early stages of DR are detected, it would be treated by laser or other therapeutic methods. In order to early detection, screening is useful [3]. In eye clinics many types of diseases related to eye are documented and diagnosed by retinal photography [4]. DR means damaging the blood vessels of the retina in the posterior part of the eye due to diabetes. DR severity can be classified into five levels, namely, no DR, mild nonproliferative DR (NPDR), moderate NPDR, severe NPDR and proliferative DR (PDR) [5]. An international clinical DR disease severity scale is shown in Table 1 [6]. Ophthalmologists use information of this table for grading DR. The determination of DR severity is important in treating the disease.

There are various techniques for automatic grading of DR. These systems use one or more features such as blood vessels, exudates (EXs), micro-Aneurisms (MAs), and texture [6–14].

Yun et al. [12] classified normal, mild NPDR, moderate NPDR, severe NPDR, and PDR stages by feed-forward neural network. The features were area and perimeter of blood vessels of color fundus images. Ahmad et al. [6] reported the use of mean, std, median features of pixels related to foveal avascular zone (FAZ) with Bayesian classifier to determine DR stages. Kahai et al. [8] applied image preprocessing, morphological processing techniques and texture analysis methods to detect the features such as area of hard EXs, area of the blood vessels, and the contrast. These features are then used as an input to the neural network for an automatic classification. Vallabha et al. [13] used a method based on global image feature extraction to classify Mild NPDR and severe NPDR. In that method the vascular abnormalities are detected using scale and orientation selective Gabor filtersbanks. Priya and Aruna [14] fed extracted features such as area, mean, and standard deviation to support vector machine (SVM), in order to classify DR color images into 3 stages (normal, NPDR and PDR).

This work describes a new method for automatic grading of 3 main stages of DR. The proposed algorithm uses fundus fluorescein angiography (FFA) and color fundus images simultaneously. MAs appeared like white small dots in FFA and they are more distinguishable than in color fundus images [15]. On the other hand, EXs are better shown in color fundus images. On this base, a fully curvelet based method is used for extraction of main objects in both FFA and color fundus images such as optic disk (OD), vessels, and FAZ. In addition to extracted features from these objects for DR grading such as FAZ enlargement and regularity, the main lesions appeared in DR such as EXs and MAs are detected using curvelet-based techniques and appropriate features are extracted from them. The main reason of using digital curvelet transform (DCUT) [16] is its ability to detect 2D singularities. In fact although wavelet transform is a powerful tool for 1D signal processing, but it does not keep its optimality for 2D signal processing because it is only able to detect 1D singularities. On this base, DCUT is an appropriate tool for separating various objects in images based on dividing the image to several subimages in various scales and orientations. For example, by amplifying the selected coefficients in proper subimages and reducing other coefficient and using other tools in curvelet domain the noise and unwanted objects can be removed and the desired object is detected [16–19].

The main setup of the proposed algorithm in this paper is as follows. In Section 2.1 preprocessing of both FFA and color retinal image are described.  Section 2.2 explains about optic disk (OD) detection based on DCUT on FFA image.  Section 2.3 is about EX detection from color image by DCUT. In Section 2.4 vessels are extracted by performing DCUT on FFA image.  Section 2.5 describes a method for segmenting FAZ based on DCUT and morphological procedures. In Section 2.6 a new method for detecting MAs in FFA images is detailed. Sections 2.7 and 2.8 describes the extracted features, and classification of these features into 3 grades (normal DR, mild NPDR + moderate NPDR, severe NPDR + PDR) using SVM. Experiments and results are given in Section 3. Finally, Section 4 provides conclusion.

## 2. Methodology

**Figure 1:**
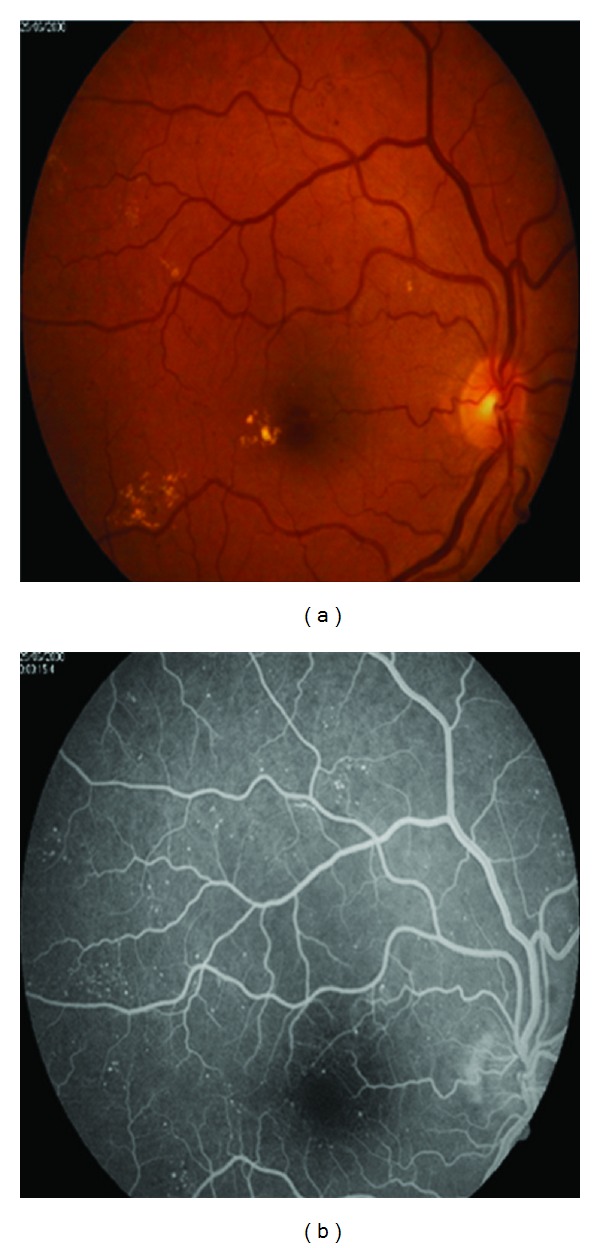
(a) EXs in color fundus image. (b) MAs in FFA image of same fundus.

**Figure 2:**
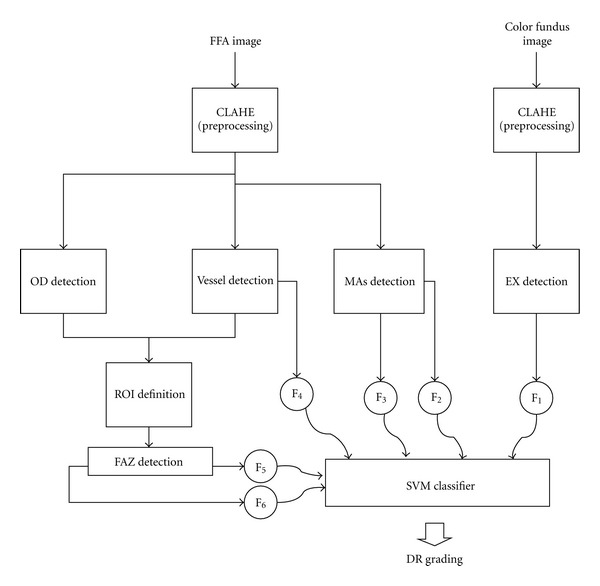
The block diagram of DR grading system. F_1_ to F_6_ indicate the features (as defined in Figure 9).

We have attempted to work on database that has both FFA image and color fundus image in this DR grading system. Because bright lesions (EXs) appeared better in color image (Figure 1(a)) but dark lesions (MAs) are more distinguishable in gray-level FFA image (Figure 1(b)). The size of the fundus images is 576 × 720 pixels. We have collected retinal image of 70 patients of different DR stages. So we have 70 FFA images and 70 color fundus images (these data are available at http://misp.mui.ac.ir/data/eye-images.html). The proposed method in this paper for DR grading is concluded in block diagram of Figure 2.

In our previous work [15], we have found FAZ location on images of DR by applying DCUT [16] on FFA image. Furthermore, the appropriate features of segmented FAZ and rate of abnormalities such as EXs and MAs are extracted and fed to SVM in order to recognize the stages of DR.

### 2.1. Preprocessing

**Figure 3:**
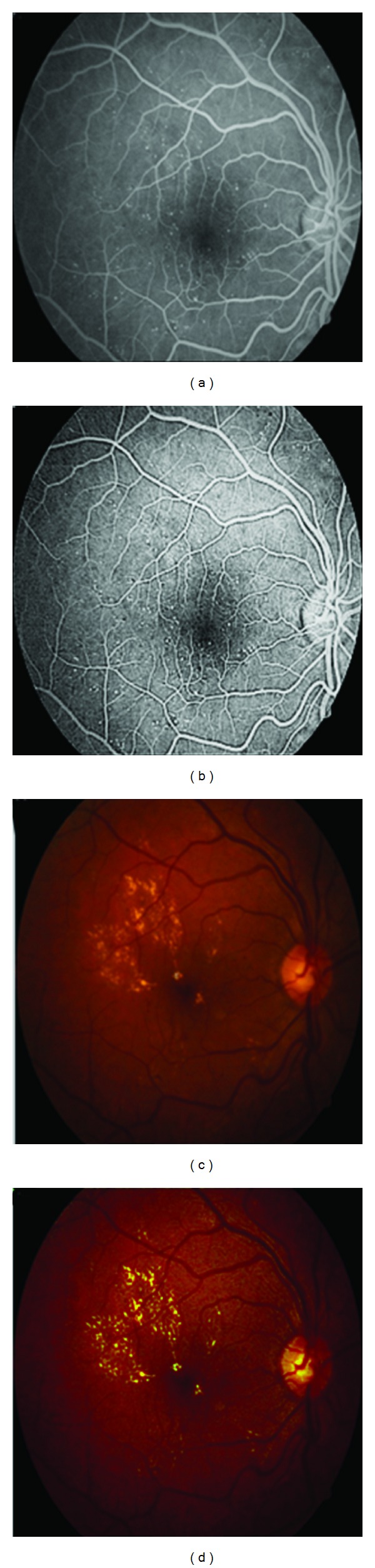
(a) The original FFA image, (b) FFA image after CLAHE, (c) the original color image, (d) the color image after CLAHE.

First of all we use contrast limited adaptive histogram equalization (CLAHE) algorithm [17, 20] and illumination equalization. So resulted image would have uniform background and high contrast.  Figure 3 illustrates CLAHE performed on both color and FFA images.

### 2.2. OD Detection

We use the presented method in [15] for OD detection. The OD might be mistaken as EXs if it is not segmented and masked out. In [18], DCUT is applied on color retinal image for detecting OD.

DCUT is a digital version of curvelet transform. The main motivation for using curvelet transform is dealing with 2D singularities such as edges in images instead of point singularities in 1D signals. In fact one of the desire properties of an appropriate 2D transform for image processing is directional selectivity (DS). DS is not a required property for 1D signals and so wavelet transform is nearly an optimum choice for 1D signal processing. However for high dimensional data DS plays a key role and on this base ridgelet transform [16] was introduced using the same idea used for wavelet transform (i.e., in wavelet transform a 1D finite basis function is used for producing all basis functions by dilation and scaling, similarly in ridgelet transform a 2D finite basis function is used for producing all basis functions by dilation, scaling and *rotation*). Note that ridgelet transform is only able to detect straight lines (a special kind of 2D singularities) while we need to deal with all kind of edges in an image. To solve this problem applying ridgelet transform on various subbands of a multiscale transform was suggested. This idea that leads to curvelet transform cause detecting small straight lines and connecting these small lines can detect approximately any desire curve and edge in an image. 

Here we use the proposed DCUT-based OD detection method in [18] for FFA gray level images [15]. For this reason after applying DCUT on FFA image, the curvelet coefficients are modified with exponent of 5. To segment candidate regions, Canny edge detector is used to detect the initial boundaries and then some morphological operators are employed for final detection. (The edges are *dilated* using a flat, disk-shaped structuring element with radius 1 pixel, then the holes are *filled* in order to remove the inappropriate regions, and finally the image is *eroded* to get location of candidate region of OD.) 

**Figure 4:**
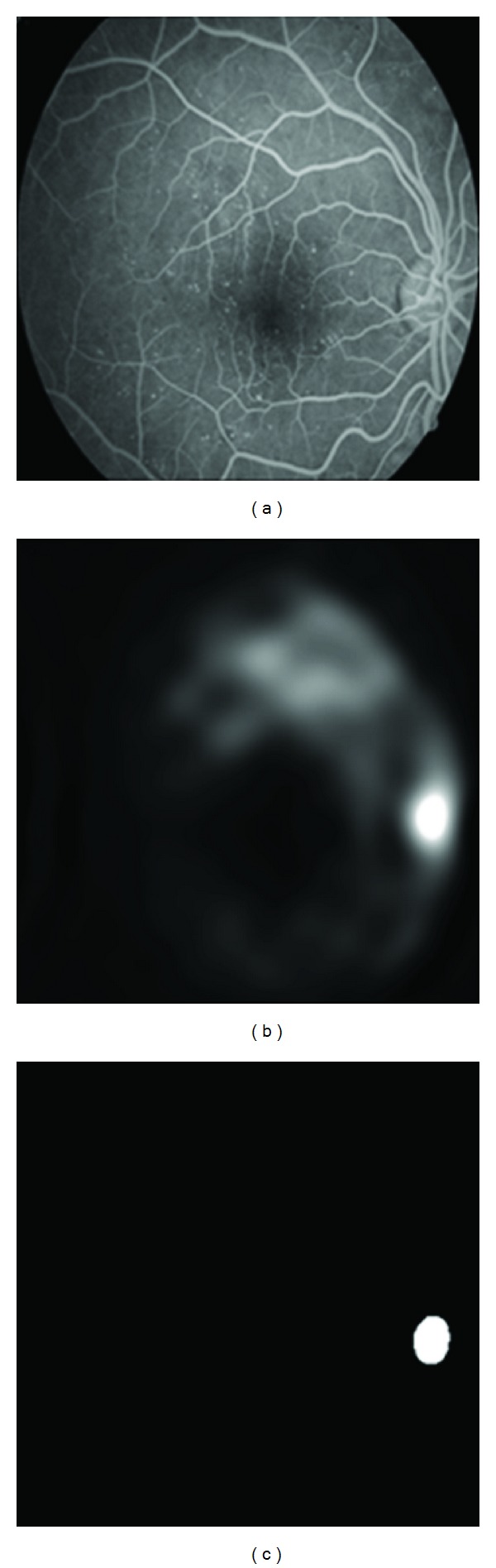
(a) The original FFA image, (b) the image after amplifications of bright objects by DCUT, (c) localization of OD after applying Canny edge detector and morphological operators [15].

Finally the OD is extracted based on this fact that OD is partly covered with vessels.  Figure 4 shows this procedure for proposed FFA image in Figure 1.

### 2.3. Exudates Detection

EXs are detected by performing DCUT on color fundus images. The main steps of EXs detection are presented here.

**Figure 5:**
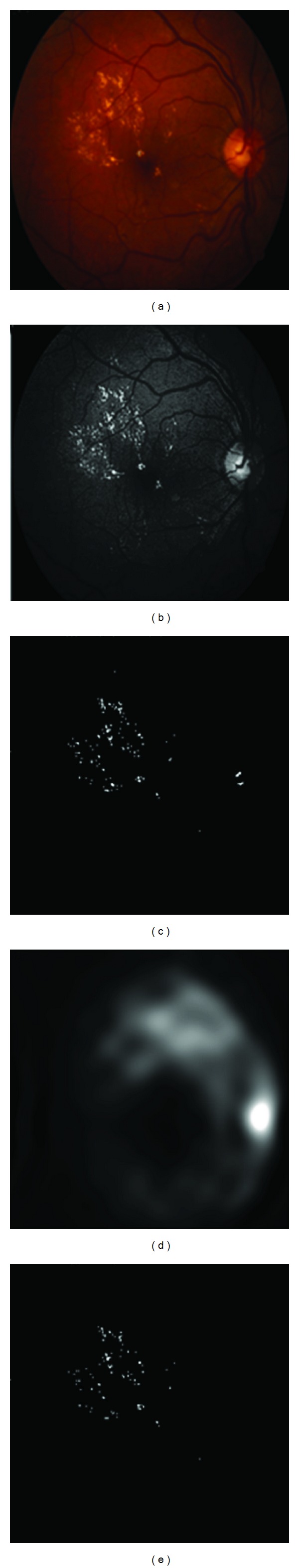
(a) The original image, (b) reconstructed image after modifying DCUT coefficients, (c) the produced image after thresholding, (d) OD localization, (e) removing OD and extracting EXs.

- Enhancement of bright lesions by applying DCUT on enhanced image and modifying its coefficients.
- Extracting of candidate regions for EXs by thresholding (Figure 5(c)).
- Removing of OD (Figure 5(e)).

In order to improve the contrast of EXs, intensity of gray levels in green channel (*f*(*i*, *j*)) is changed as follows:

(1) $$\begin{array}{c} g\left(i,j\right)=9\times f\left(i,j\right)-9\times \tilde{f}w+90. \end{array}$$

$\tilde{f}w$ is average intensity in a window of 3 × 3 (Figure 5(b)). So instead of green channel of RGB image, this enhanced gray level image is used and then DCUT is performed on new color image for extracting EXs (Figure 5).

### 2.4. Vessel Detection

Again we use DCUT for segmenting vessels [15]. For detecting vessels the following steps are proposed.

(1) Inverting FFA image.
(2) Curvelet-based contrast enhancement.
(3) Taking DCUT of the match filtered response of enhanced retinal image.
(4) Removing low frequency component, and amplifying all other coefficients.
(5) Applying inverse DCUT.
(6) Thresholding using the mean of the pixel values of the image.
(7) Applying length filtering and removing misclassified pixels.
 Actually the cross-section of retinal vessels has a Gaussian shaped intensity profile [19]. On this base, the following filter is convolved with the original image in order to amplify the blood vessels:
  (2) $$\begin{array}{c} f\left(x,y\right)=-\exp\left(-\frac{x^{2}}{2\sigma^{2}}\right)\;\text{for}\;\;y\leq L/2, \end{array}$$
  where *L* is the length of a segment of vessel with a fixed orientation. The negative sign in the Gaussian function indicates that the vessels are darker than the retinal background as in FFA. *y*-axis is the direction of the vessel and since a vessel may be oriented at any angles, this kernel is applied in every 15° (12 different directions), and the maximum value in each pixel is extracted.

**Figure 6:**
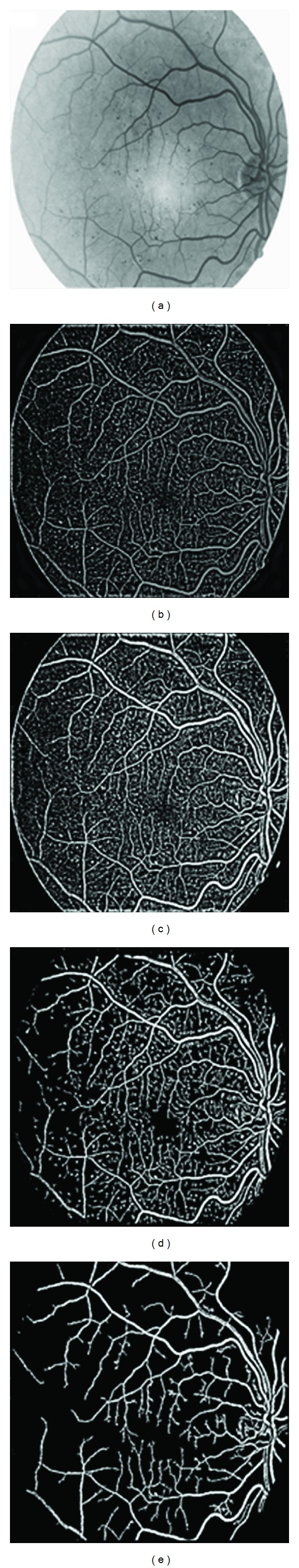
(a) Inverse of FFA, (b) matched filter response of enhanced image, (c) high frequency component of image, (d) the produced image after thresholding, (e) the extracted vessels by applying length filtering.

Figure 6 shows the produced images from above steps for proposed FFA image in Figure 1.

### 2.5. FAZ Detection

**Figure 7:**
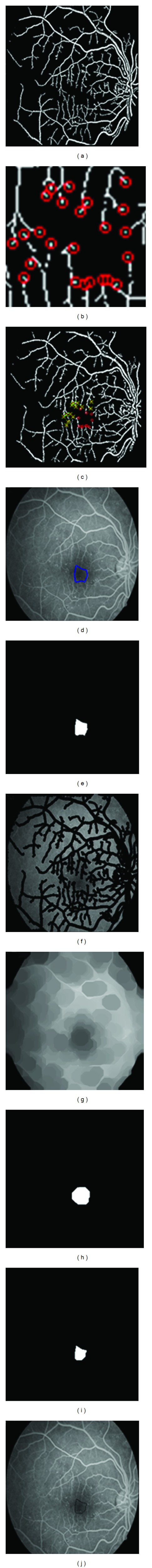
(a) Vessel mapped image, (b) showing end-points in predefined ROI, (c) removing end-points which are far from center of FAZ, (d) connecting selected end-points, (e) extracted FAZ from DCUT method, (f) subtracting vessels from original image, (g) applying morphological closing, (h) extracted FAZ by thresholding (g), (i) applying logical and between extracted FAZ in (e) and (h), (j) showing the final FAZ region.

As completely discussed in [15, 21], by detecting end points of extracted vessels in defined ROI, and connecting these points, FAZ region is segmented (Figure 7). For this reason the following steps are proposed.

1. Vessel extraction based on DCUT.
2. OD extraction based on DCUT.
3. ROI definition based on this fact that macula locates on 2.5 OD diameters away from center of OD.
4. Finding end-points of vessels in ROI region. For this reason the following steps are proposed.
  - A 3 × 3 window is used for each pixel in ROI. If the summation of intensities in this window is 2, this pixel is selected as an end-point. (Smaller values correspond to no connectivity such as a single white pixel or a black area and grater values correspond to within vessel areas.)
  - The center of these end-points is obtained by averaging all end-points' coordinator and then the average distance of all end-points to this center is calculated. The final end-points are selected by comparing each end-point's distance against this mean value and discarding end-points that their distances are greater than mean value.
5. Connecting selected end points to each other.

### 2.6. MAs Detection

**Figure 8:**
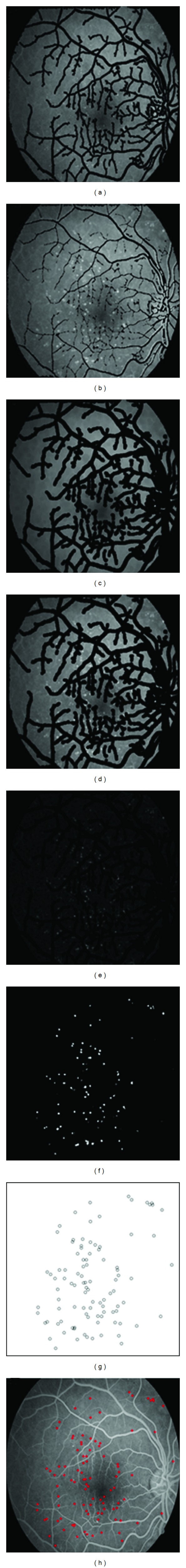
(a) Original image after removing vessels, (b) applying morphological dilation on (a), (c) background image by applying morphological erosion on (b), (d) mapping zero pixels of (c) on (b), (e) removing background, (f) produced image after thresholding, (g) detected MAs, (h) MAs on original image.

MAs are the earliest clinical sign of DR. In this paper, a new method for detecting MAs is presented. In order to detect MAs, segmented vessels, are subtracted from original enhanced FFA image (Figure 8(a)). Then morphological dilation is applied on resulted image (Figure 8(b)). In this image, MAs appear brighter than other pixels. By applying morphological erosion on this image, we could reach to background of retina (Figure 8(c)). In the next step the background is removed from dilated image (Figure 8(e)). After removing background, bright small regions are enhanced and finally MAs are detected by thresholding (Figure 8(f)). Since the only bright objects in Figure 8(e) are MAs using a simple threshold such as 0.1 maximum of intensity (e.g., for a 8-bit image it can be set to 26) is used in thresholding step.

### 2.7. Feature Extraction

In order to classify different stages of DR, we must extract appropriate and significant features. The feature set should be selected such that the between-class discrimination is maximized while the within-class discrimination is minimized. In this section, we explain about the selected features for DR grading.

(1) Area of detected FAZ: In [22] it has been shown that the area of FAZ is changed relative to the stage of DR.
(2) Circularity of detected FAZ: FAZ region is an oval shape in normal retinal images. So stage of DR could have great influence on shape of this region. Analyzing variance of distance between points around FAZ and center of FAZ could be a good feature for DR grading.
(3  &  4) Total number of MAs and number of MAs in FAZ boundary: As shown in Table 1 the number of MAs is very important in grading DR. Also the position of lesions such as MAs relative to the macula is another useful feature for analysis and classification of DR.
(5) Total area of EXs: The higher stage of the DR would have more EXs due to damages or leakages of the blood vessels.
(6) Area of blood vessels: In some higher stages, main blood vessels are damaged (especially around macula) and new vessels are created (Neovascularization). These new vessels are thin. So, higher stages have fewer blood vessels because of damage.

### 2.8. Classification

In the last step SVM is used to classify DR severity. This grading is based on extracted features from: (1) anatomical structures such as FAZ region and vessels and, (2) lesions which appeared due to DR such as EXs and MAs. 

SVM isasetofrelated supervisedlearningmethodsusedfor grading [21, 23, 24]. In factthe SVM separates 2 classes based on a function which is induced from training database. The SVM construct different hyper planes. As the goal is reaching to maximum margin, optimal hyper plane is selected [25]. The margin is distance between classifier and the nearest data point of each class [24]. The points that lie closest to the optimum hyper plane are called support vectors. 

In this paper, we apply SVM twice. First one is for separating grade1 (normal) from grade 2 (mild and moderate NPDR) and grade 3 (severe NPDR and PDR). Second one is separating grade 2 and grade 3. 

## 3. Results 

**Figure 9:**
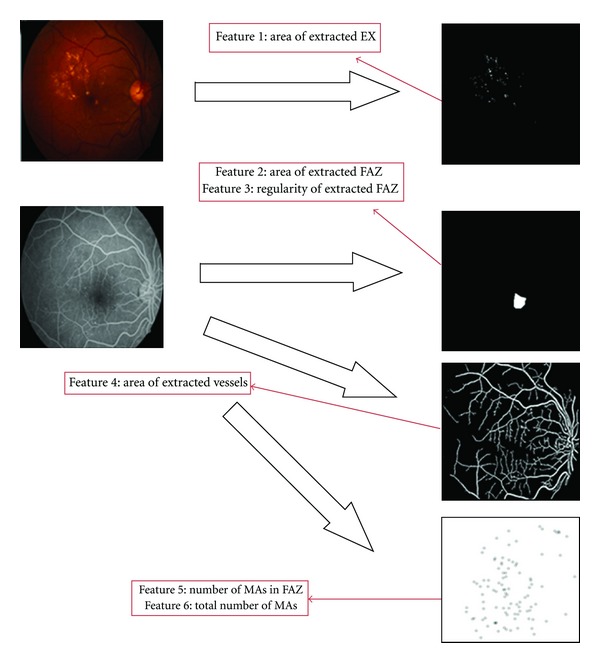
Extracted features from color and FFA images.

As we have shown in block diagram of Figure 2, we need some appropriate features to classify DR patients. These features, which are discussed completely in the previous section, are extracted from both color retinal image and FFA retinal image (Figure 9). Note that the proposed grading methods are mostly based on only using color fundus images. However in this paper, we collect both FFA and color fundus images. Note that any publicly available database from both FFA and color fundus images does not exist and so we uploaded our own data to http://misp.mui.ac.ir/data/eye-images.html. 70 patients were involved in this work. The presented study classifies DR into 3 groups. First group is normal stage. In the second group the mild and the moderate stages are grouped together, and the third one is related to higher stages where severe NPDR and PDR are grouped together. Extracted features of different stages of DR are shown in Table 2. Our database includes respectively 30, 25, and 15 images for first, second, and third groups and 30% of data are used for training and the others for test. We also changed training and test data and averaged the results. As explained before, at first the features are fed to SVM to classify the data to two groups: grade 1 and grade 2 + grade 3. If data does not belong to grade 1, the features are again fed to another SVM to classify the data to grade 2 and grade 3. This grading algorithm has sensitivity and specificity of 100%. As we can see from Table 2, it is clear that the (mean of) proposed features are significantly far from each other and it is the main reason of error-free performance of this system.

## 4. Conclusion

In this paper, a curvelet-based algorithm for DR grading was introduced. In this algorithm it is necessary to detect OD, vessels, FAZ, EXs, and MAs that all of them are detected by employing curvelet transform. In the next step 6 features were obtained from extracted FAZ and detected lesions and used as an input vector for SVM classifier. This algorithm was able to completely distinguish between “normal Stage”, “mild/moderate NPD”, and “severe NPDR/PDR”. 

As an extension of this study, it is suggested to extract more features to increase the ability of algorithm for grading all stages of DR. This stage needs collecting more data (including both FFA and color fundus images) for each DR grade. 

In this paper, only curvelet transform is used as an oriented transform that is able to separate the image to several subimages with specific time-frequency and orientation components. Other directional transforms such as dual tree complex wavelet transform [26], contourlet transform [27], steerable pyramid [28], and shearlet transform [29] can be substituted with curvelet transform.

## Figures and Tables

**Table 1:** International clinical DR severity scale [5, 6].

Severity level	Findings observable
No DR	No abnormalities

Mild NPDR	This is the earliest stage of retinopathy and vision is usually normal except in some cases. Small swellings known as micro-aneurysms or flame-shaped hemorrhages start to develop in the fundus quadrants

Moderate NPDR	There will be micro-aneurysms or hemorrhages of greater severity in one to three quadrants and leakage might occur, resulting cotton wool spots and exudates and so fourth to be present in the retina

Severe NPDR	Any of the following: (i) Severe (more than 20) hemorrhages and MAs in all four quadrants of the fundus (ii) Definite venous beading in at least two quadrants (iii) Severe damage to the small blood vessels in at least one quadrant but no signs of any PDR

PDR	One or more of the following: Neovascularization Vitreous/preretinal hemorrhage

**Table 2:** Average of extracted features in different stages.

	No DR	Mild/Moderate NPD	Severe NPDR/PDR
No. data	30	25	15
Area of EXs	15	450	2987
No. of MAs in FAZ	0	13.8	15.04
Total no. of MAs	0.94	110	73.76
Area of vessels	52567.14	45654	29521
Area of FAZ	3498.25	5773	13624
Variance	12.93	27.07	110.84
